# BMAL1 promotes colorectal cancer cell migration and invasion through ERK‐ and JNK‐dependent c‐Myc expression

**DOI:** 10.1002/cam4.5129

**Published:** 2022-08-10

**Authors:** Lina Shan, Wenqian Zheng, Bingjun Bai, Jinghui Hu, Yiming Lv, Kangke Chen, Xiaowei Wang, Yangtao Pan, Xuefeng Huang, Hongbo Zhu, Sheng Dai

**Affiliations:** ^1^ Department of Colorectal Surgery Sir Run Run Shaw Hospital, School of Medicine, Zhejiang University Hangzhou China; ^2^ Key Laboratory of Biotherapy of Zhejiang province Hangzhou China

**Keywords:** BMAL1, c‐Myc, colorectal cancer, EMT, MAPK signaling pathway

## Abstract

**Background:**

Cancer metastasis is still a life threat to patients with colorectal cancer (CRC). Brain and muscle ARNT‐like protein 1 (BMAL1) is an important biological proteins that can regulate the behavior of cancer cells and their response to chemotherapy. However, the role of BMAL1 in the tumorigenic phenotype of CRC remains unclear. Here, we aim to investigate the functional role and mechanisms of BMAL1 in CRC.

**Methods:**

The mRNA expression of BMAL1 was studied using the Cancer Genome Atlas (TCGA) databases. The protein level in clinical tissues was confirmed by immunohistochemistry (IHC). The effects of BMAL1 on the epithelial‐to‐mesenchymal transition (EMT) and proliferation of CRC cell lines (including BMAL1 overexpressed or silencing cells) were studied by Transwell, wound healing, CCK‐8 and colony formation experiments. A series of experiments were conducted to demonstrate the mechanisms of BMAL1 regulating EMT and cancer proliferation in vitro and in vivo.

**Results:**

We found that BMAL1 expression was closely related to the poor prognosis of CRC. BMAL1 overexpression promoted cell proliferation and migration. Mechanistically, we found that BMAL1 may activate the epithelial‐to‐mesenchymal transition (EMT) pathway and induce the β‐catenin release further promotes the expression of oncogene c‐Myc and the migration of colorectal cells by activating MAPK pathway. However, BMAL1 silencing achieved the opposite effect. In addition, blocking MAPK‐signaling pathway with specific inhibitors of ERK1/2 and JNK can also downregulate the expressions of c‐Myc in vitro. Taken together, these results suggested that the BMAL1/ c‐Myc‐signaling pathway may regulate the metastasis of CRC through the JNK/ERK1/2 MAPK‐dependent pathway.

**Conclusions:**

Our study showed that BMAL1 promotes CRC metastasis through MAPK‐c‐Myc pathway. These results deepen our understanding of the relationship between BMAL1 and tumorigenic phenotypes, which may become a promising therapeutic target for BMAL1 overexpressing CRC.

## Graphical abstract

Our study demonstrate that BMAL1 plays a critical role in cell migration and invasion of CRC cells via MAPK‐c‐Myc pathway. Our results may deepen our understanding in the relationship of BMAL1 and tumorigenic phenotypes and be developed into a promised therapeutic target for the BMAL1‐overexpression CRC.

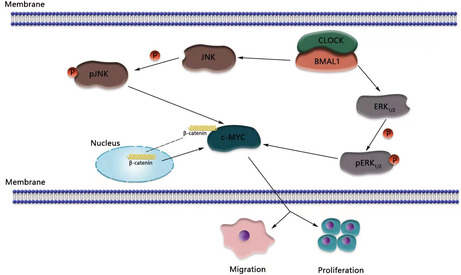

## 1 BACKGROUND

CRC is one of the most aggressive cancers in European and American countries. The incidence and mortality rate of CRC also have an increasing trend in China.^1^, ^2^ CRC is a common and complex disease caused by genetic and environmental factors and their interactions. As the early symptoms of CRC are not obvious, tumor metastasis is common at diagnosis and during follow‐up. It is well known that distant invasion and metastasis are adverse prognostic factors leading to CRC‐related mortality. In recent years, there has been no obvious breakthroughs in surgical technology or molecular targeted therapy. Therefore, searching for effective methods and key pathogenic mechanisms are crucial for improving colorectal cancer management.^3^, ^4^, ^5^

BMAL1, one of the important biological clock proteins in mammals, which is located on human chromosome 11.^6^, ^7^ Recently, many studies have shown that the biological clock system plays an important role in cancer cell proliferation, apoptosis, growth, metabolism, and tumor treatment.^8^ Circadian rhythm may regulate the expression of various genes in multiple cell types, and its disruption may increase the risk of cancer. As an important core clock protein, BMAL1 is closely related to tumorigenesis. Karantanos T and colleagues proposed that the expression of BMAL1, a key clock gene, is more common in colorectal carcinomas, but less common in colorectal adenomas.^9^ Additionally, circadian rhythm genes might substantially influence the efficacy and toxicity of some antitumor drugs.^10^, ^11^, ^12^ According to a recent study, BMAL1 may play a role in acquisition chemotherapy and targeted treatment resistance in patients with CRC.^13^ Based on this evidence, we hypothesized that BMAL1 may contribute to tumorigenesis and metastasis of CRC. The study of molecular events driving metastasis is crucial to reveal the mechanisms involving tumorigenesis and metastasis in CRC.

One of the key events of CRC invasion and metastasis is epithelial‐to‐mesenchymal transition (EMT).^14^ In the process of cell migration, polarized epithelial cells lose their cell polarity and intercellular connection, and obtain the characteristics of mesenchymal cell. The cancer cells become more invasive so as to result in metastasis. This event is defined as the epithelial‐to‐mesenchymal transition. Recent study have found that BMAL1 was a CRC‐related gene and was associated with poor prognosis. Gain‐ and loss‐of function analyses revealed that BMAL1 play a role in inducing proliferation and metastasis of colorectal cancer cells both in vitro and in vivo. Further functional experiments indicated that BMAL1 induces CRC progression and maintains the EMT phenotype via the MAPK (ERK1/2 and JNK)/c‐Myc pathway in CRC. These findings provide a new feasible target for the colorectal cancer treatment.

## 2 MATERIALS AND METHODS

### 2.1 Analyses of TCGA and public gene expression datasets

The datasets generated and analyzed during the current study are available in the TCGA database (https://www.proteinatlas.org) (ENSG00000133794). We obtained the RNA sequencing data and paired clinical information of CRC samples from the TCGA database. Then, we used the cut‐off value recommended by the website for separating the high and low levels of BMAL1 expression in the survival analysis and performed a survival analysis to determine the correlation between BMAL1 mRNA expression and the prognosis of the 434 cases with colon adenocarcinoma.

### 2.2 Patients and samples

The tissue samples including tumor and corresponding normal tissues were collected from 84 patients disgnosed with CRC by Sir Run Run Shaw Hospital of Zhejiang University School of Medicine. No other malignancies were observed in these patients. The written informed consent of patient and the approval from the Institutional Research Ethics Committee were obtained.

### 2.3 Cell culture and transfections

RKO, HCT116, and SW480 colorectal cancer cell lines were purchased from the American Type Culture Collection (ATCC) and cultured in RPMI1640 or DMEM containing 10% FBS. Ubi‐MCS‐3FLAG‐SV40‐EGFP‐IRES‐puromycin‐BMAL1 expression lentiviruses, hU6‐MCS‐Ubiquitin‐EGFP‐IRES‐puromycin BMAL1 inhibitor lentiviruses and their corresponding negative controls were purchased from Shanghai Genechem Co. These lentiviruses were transfected to establish stable cell lines. After 10 days of culture with puromycin, the stably expressed cell lines were selected. After selection, stably expressed cell lines were harvested and confirmed using qRT–PCR and Western blotting assays.

### 2.4 Immunohistochemistry

BMAL1 Immunohistochemistry (IHC) assay was based on the previous studies.^15^ In short, the BMAL1 expression of clinical tissues was assessed by IHC analysis using anti‐BMAL1 antibody (1:200; Proteintech). The primary antibody was cultured with tissue sections overnight at 4°C. The final evaluation criteria depend on the percentage of positive cells and the degree of staining. The staining intensity score was defined and classified as negative, weak, moderate, or high‐strong, which were counted as 0, 1, 2, and 3, respectively. The cell proportion score was defined as follows: <10% = 0, >10% to 25% = 1, >25% to 50% = 2, >50% to 75% = 3, and >75% = 4. Finally, these two scores were then calculated by multiplying. High BMAL1 expression: score > 4; low BMAL1 expression: the final score was ≤4. The operation and the scoring of the c‐Myc staining were the same as BMAL1.^16^ If the final score was ≥4, the sample was considered c‐Myc‐positive.

### 2.5 Western blotting

Total‐cell protein was extracted using RIPA buffer with protease inhibitors and phosphatase inhibitor (Life Technologies). The BCA Protein Assay Kit (Beyotime Bio‐ technology) was used to quantify the protein concentration. An equal amount of protein was loaded onto 8%–12% SDS‐PAGE and then transferred to PVDF membrance (Hercules bio rad). After incubation with 5% skimmed milk at room temperature for 1 h, the membrance was incubated with the following antibodies: anti‐BMAL1, anti‐E‐cadherin, anti‐N‐cadherin, anti‐vimentin, anti‐β‐catenin, anti‐phospho‐MEK, anti‐phospho‐ERK1/2 (Cell Signaling Technology), anti‐p38, anti‐JNK, anti‐c‐Myc, anti‐RAF, and anti‐Ki67 (Abcam). Anti‐GAPDH mouse monoclonal antibody (Cell Signaling Technology) was used as load control. The ECL system was used for blots (Amersham Biosciences).

### 2.6 Colony formation assay of cell proliferation

A total of 500 transfected cells were incubated in 6 cm dishes for 10 days. Then, cell clones were fixed with 10% formaldehyde for 5 minutes, stained with 1% crystal violet and counted with an optical microscope. Each assay was performed in triplicate.

### 2.7 Wound healing assay and invasion assay

For the wound healing test, different groups of 1 × 10^6^ RKO, HCT116 and SW480 cells, and cells were cultured to near 90% confluence in the plates. Then scratched with a sterile 1 ml pipette tip. After the damaged cell layer was washed, it was cultured in complete medium without FBS. The wounds were photographed with a light microscope at 0 and 24 h.

Transwell cell migration plates (Corning Incorporated) were used for invasion and migration assays. Transwell migration experiments were carried out using Corning 8‐μm chambers according to the manufacturer's instructions (BD Biosciences). For the invasion assay, cancer cells (5 × 10^4^ cells/well) from different groups (RKO/Control; RKO/BMAL1;RKO/shControl; RKO/shBMAL1; SW480/Control; SW480/BMAL1; SW480/shControl; SW480/shBMAL1) with 200 μl serum‐free medium were placed into the upper chamber, with 600 μl medium supplemented with 10% FBS in the bottom chamber. After 36 h, the migrated cells in the underside of the membrance were immersed and washed with PBS, fixed with 4% paraformaldehyde, stained using 0.1% crystal violet, washed three times with water, and counted under light microscopy.

### 2.8 Xenograft tumor model

Four‐week‐old Male BALB/c nude mice were placed in an animal facility for 12‐h light on–off cycle at consant room temperature, and then the animals were randomly divided into four groups (6 mice per group). BALB/c nude mice were subcutaneously injected with 5 × 10^6^ viable cells suspended in 200 μl PBS in the right flank. The size of subcutaneous tumor was measured with Vernier calipers on Days 10, 15, 20, 25, 30, and 35. The tumors were dissected and embedded in paraffin after 35 days. The final volume of tumor tissues was calculated using the following equation: tumor length (mm) = (tumor length + tumor width)/2. The tumors were also IHC stained with antibodies against BMAL1, E‐cadhrein, and c‐Myc. Animal studies were approved by the Animal Care Institutional and Use Committee of Sir Run Run Shaw Hospital of Zhejiang University.

### 2.9 Statistical analysis

All data were analyzed using SPSS version 16.0 statistical software packages (IBM). The data are expressed as means ± standard deviations (SD) to indicate the variability of the data. Chi‐square test or Fisher's exact test were used to compare the correlation between BMAL1 and clinicopathological feactures. Statistically significant differences between groups were compared using the Student's t test. The patients' survival outcomes were evaluated according to the Kaplan–Meier Plotter and compared using log‐rank test. A two‐tailed *p*‐value <0.05 was statistically significant.

## 3 RESULTS

### 3.1 High BMAL1 expression predicted poor survival of patients with CRC

**FIGURE 1:**
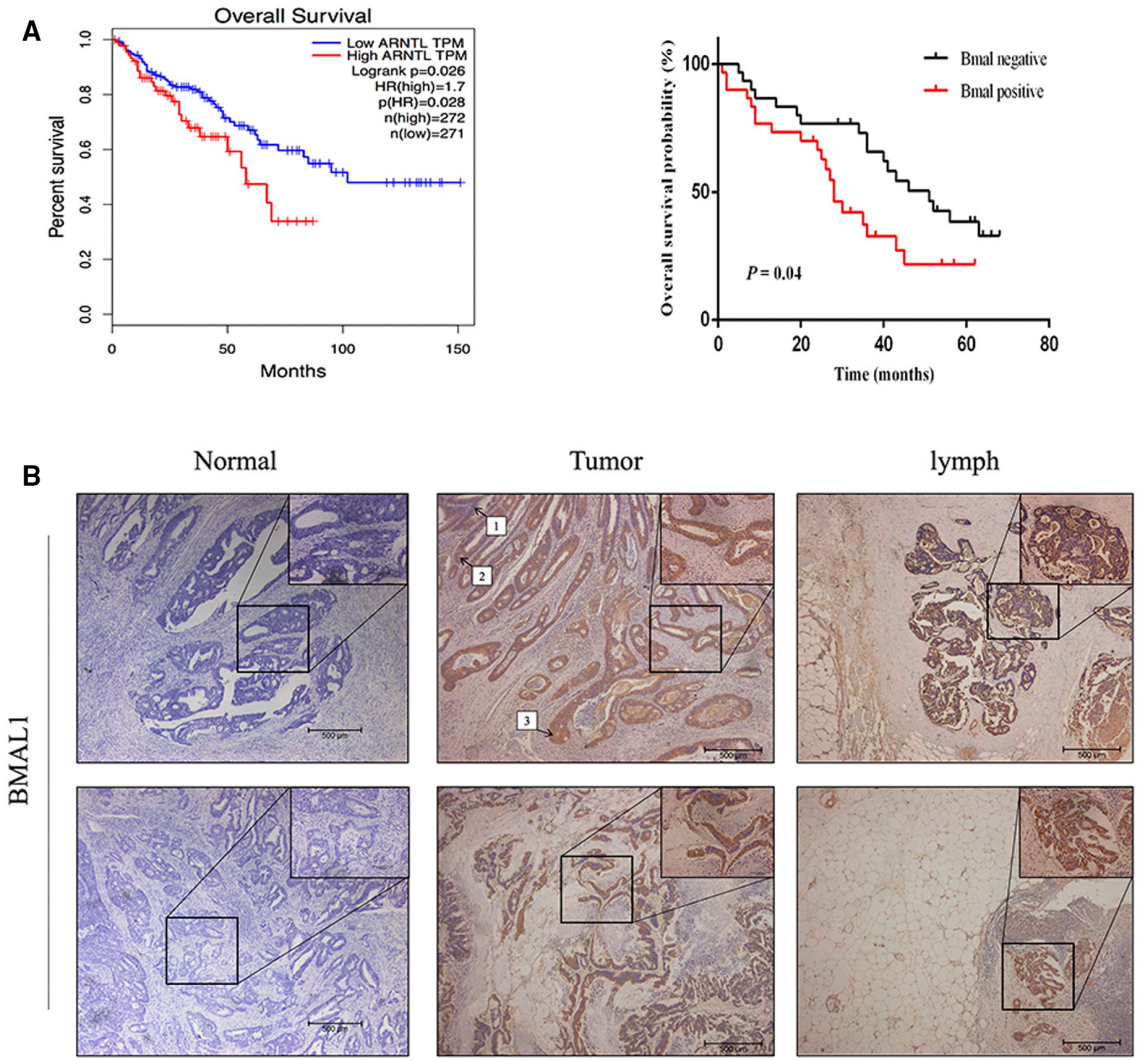
High BMAL1 expression was significantly associated with poor prognosis in CRC. (A) Patients in The Cancer Genome Atlas (TCGA) and Sir Run Run Shaw cohorts (*n* = 84) were stratified according to the BMAL1 gene expression signature. A total of 58.3% (49/84) CRC tissues were BMAL1 overexpression. Kaplan–Meyer overall survival curves of CRC with different BMAL1 genotypes (*p* < 0.05). (B) Representative photographs showing immunohistochemical expression of BMAL1 in the adjacent normal tissue (0 = negtive), colorectal cancer and the lymph node. 40× & 200× magnification; 1 = mild, 2 = moderate, 3 = strong.

We analyzed the BMAL1 mRNA information in samples from The Cancer Genome Atlas (TCGA) and studied the clinical relevance of BMAL1. Survival analysis indicated that BMAL1 expression was an independent prognostic factor in patients with CRC (*p* < 0.05, Figure 1A). At the same time, in the Sir Run Run Shaw Hospital (SRRSH) cohort, we first evaluated BMAL1 expression level in 84 primary colorectal adenocarcinoma specimens and examined the correlation between BMAL1 expression and clinicopathological parameters. The typical IHC staining of BMAL1 protein in CRC tissues were shown in Figure 1B. Tissues used were obtained from 84 patients with CRC treated between January 2012 and November 2015 in SRRSH. The baseline characteristics of the patients are summarized in Table 1. In addition, 58.3% of patients exhibited BMAL1 positive in primary CRC tumor, which was common among younger patients (*p* < 0.05). Importantly, the expression of BMAL1 played an important role in determining the prognosis of patients with CRC (*p* < 0.05, Figure 1A). However, univariate Cox regression model revealed that BMAL1 expression was not related to T stage, lymphatic invasion, tumor size or tumor location. Taken together, these results confirmed that BMAL1 was associated with CRC outcomes.

**TABLE 1:** Clinicopathological features according to BMAL1 in CRC patients

Variable	No. patients	BMAL1 expression		
		BMAL1 (+)	BMAL1 (−)	*p* value
Sex				
Male	55	31	24	0.649^b^
Female	29	18	11	
Age (years)	58.881^a^	56.551^a^	62.143^a^	**0.038** ^d^
Tumor size(mm)	42.440^a^	42.306^a^	42.629^a^	0.944^d^
yT category				
T_1_, T_2_	9	5	4	1.000^c^
T_3_, T_4_	75	44	31	
Lymphatic invasion				
Absence	23	10	13	0.090^b^
Presence	61	39	22	
Location				
Colon	55	35	20	0.175^b^
Rectum	29	14	15	

*Note*: *p* values <0.05 are in bold.

^a^ Average.

^b^ Square test.

^c^ Fisher's exact test.

^d^ *t* test.

### 3.2 BMAL1‐KD inhibited Ki67 activation and the proliferation of colorectal cancer cells

**FIGURE 2:**
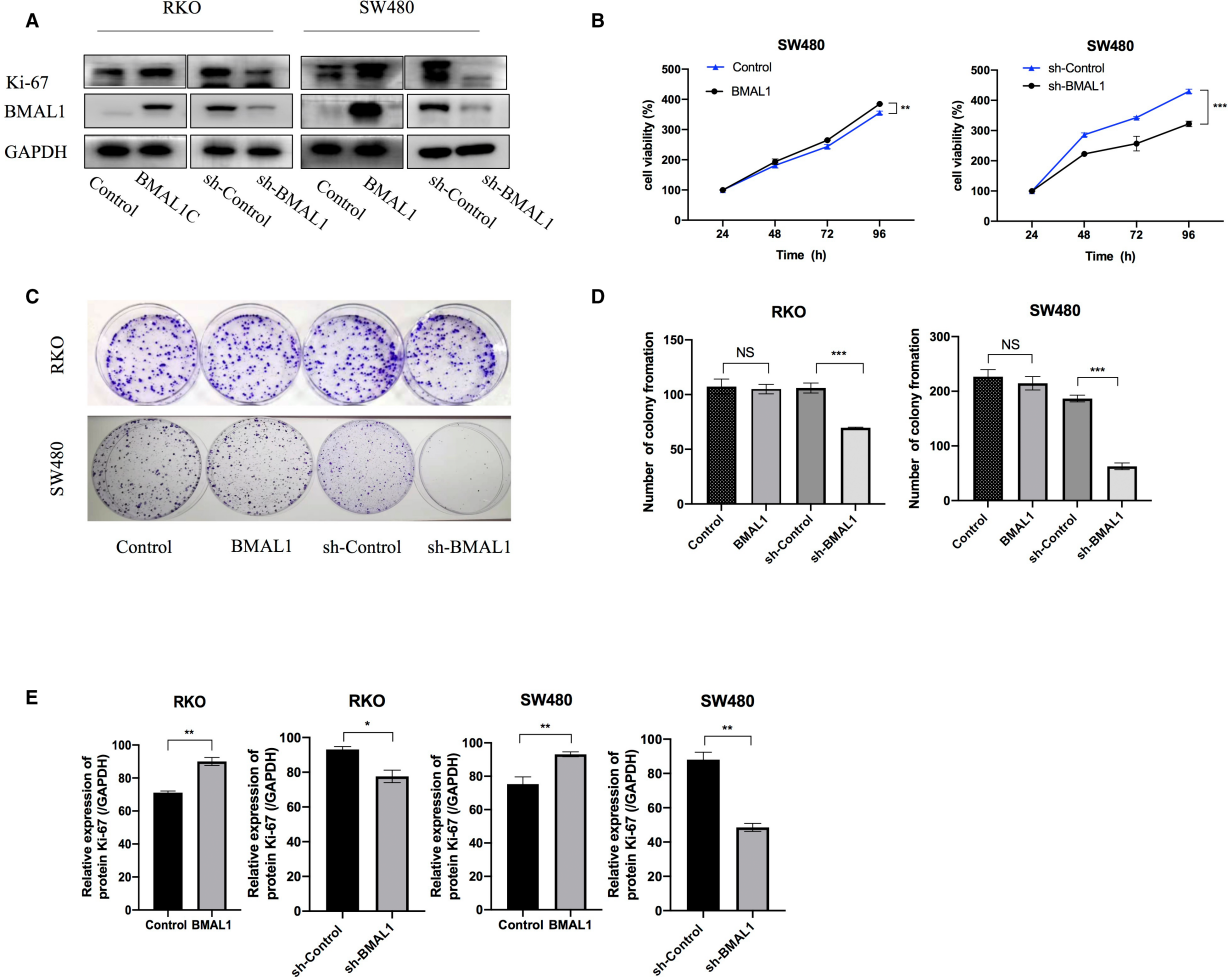
BMAL1 affects colorectal cancer cells' growth. (A) Representative western blot analysis of BMAL1 and Ki‐67 protein expression in transfected cell lines. (B) SW480 cells transfected with BMAL1 and shBMAL1 were subjected to the CCK‐8 assay after transfection. (C, D) Stably transfected RKO and SW480 cells were seeded onto 6‐well plates. The number of colonies was counted on the 10th day after seeding. (E) Western blots were quantified by ImageJ densitometric analysis and normalized to controls. Data are expressed as mean ± SD (*n* = 3). *p < 0.05, **p < 0.01 and ***p < 0.001.

Three primary CRC cell lines (HCT116, RKO, and SW480) were transfected with recombinant lentivirus in order to increase or silence BMAL1 expression in vitro and study the involvement of BMAL1 in proliferation during colorectal tumorigenesis. The overexpression or knockdown efficacy was quite sufficient, which has been verified by Western blotting (Figure 2A). Using this model, we effectively investigated the role of BMAL1 in cancer cell lines.

Here, we investigated the biological effect of BMAL1. BMAL1 expression was knocked down in RKO and SW480 CRC cell lines (RKO/sh‐BMAL1 and SW480/sh‐BMAL1) and the BMAL1‐overexpressing cell lines (RKO/BMAL1, SW480/BMAL1, and HCT116/BMAL1) were also constructed, thereby modulating different cellular activities, such as apoptosis, proliferation, cell cycle, and metastasis. The colony formation assay and MTT assay revealed that BMAL1 downregulation significantly decreased cell proliferation compared to that of control cells (*p* < 0.05, Figure 2B–D). Ki67 has been widely used as a proliferation marker to assess cell proliferation in cancer research. Western blotting analysis of Ki67 expression in colorectal carcinoma cells revealed that the level of the proliferation‐related protein Ki67 was significantly downregulated in the BMAL1 knockdown cell lines compared with the control cell lines (*p* < 0.05, Figure 2A,E). Conversely, BMAL1 overexpression significantly upregulated the Ki‐67 expression in these cell lines. Taken together, these results suggested that BMAL1 increased the proliferation of colorectal cancer cells.

### 3.3 BMAL1 promotes CRC cell metastasis via the EMT pathway

**FIGURE 3:**
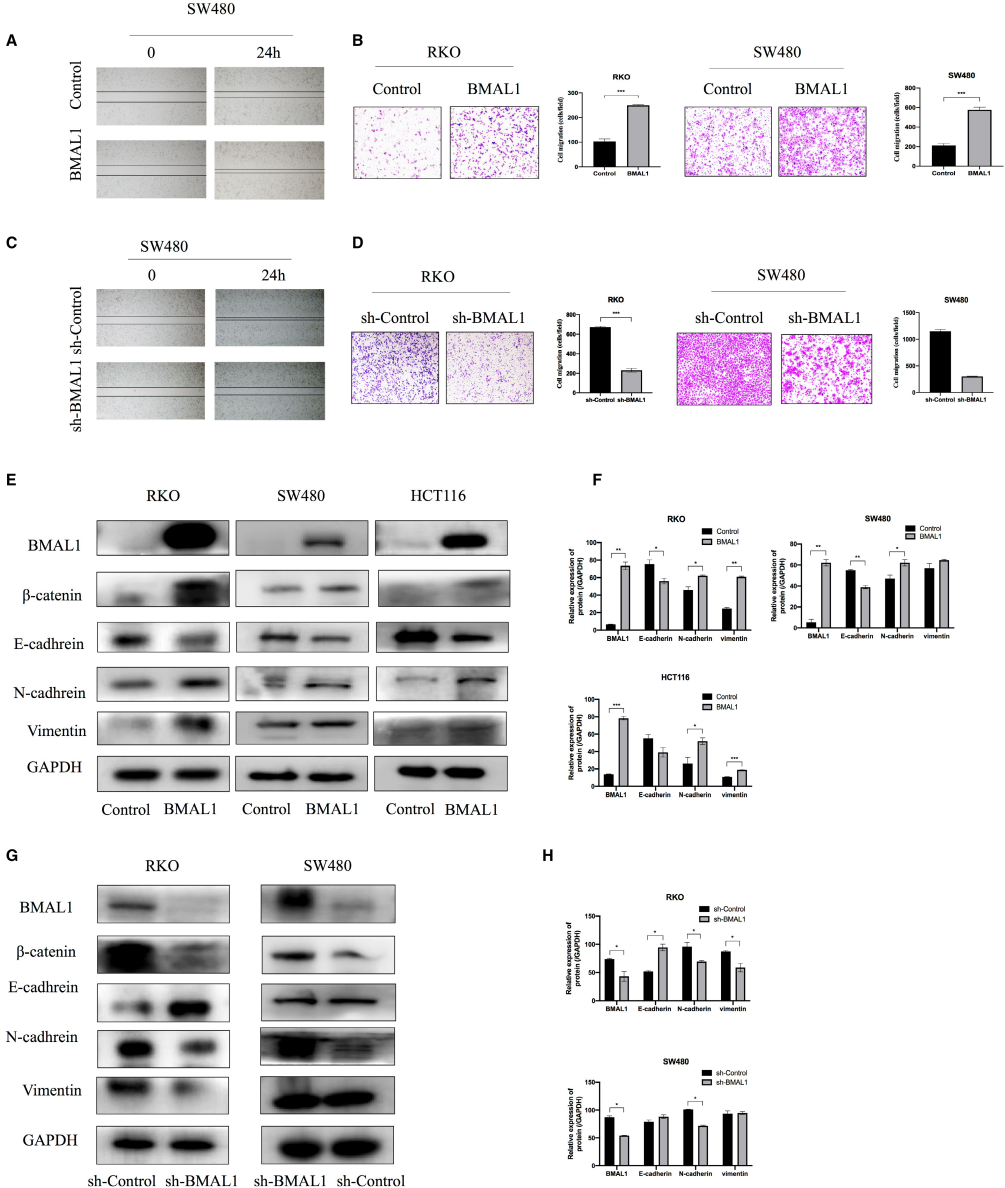
Migration activity was regulated by BMAL1. (A, C) Wound‐healing assay showed that migration activity was enhanced by BMAL1. BMAL1 silencing suppressed the migration ability of SW480 cells. (B, D) Transwell assays showed that BMAL1 enhanced the migration and invasion potential of CRC. BMAL1 silencing decreased the migration and invasion of RKO and SW480 cells. (E, G) Western blot assay showed that BMAL1 significantly increased the expression of β‐catenin, N‐cadhrein, vimentin but inhibited E‐cadhrein expression. (F, H) Western blots were quantified by ImageJ densitometric analysis and normalized to controls. Data are expressed as mean ± SD (*n* = 3). *p < 0.05, **p < 0.01 and ***p < 0.001.

We next tested whether BMAL1 affects the migration and invasion of CRC cells. Wound healing assays and Transwell assays were performed to assess the effect of BMAL1 on CRC cell motility. RKO/BMAL1 and SW480/BMAL1 cells migrated significantly quicker than the corresponding control cells (Figure 3A,B). Transwell assays showed that BMAL1 markedly promoted the migration and invasive abilities of CRC cells. The opposite results were observed in the BMAL1‐silenced cell lines (Figure 3C,D). Collectively, BMAL1 promoted CRC migration and invasion.

During the multi‐step process of malignant tumors, which are initially benign, epithelial cells acquire some obvious mesenchymal features, which makes them have the ability to locally spread to adjacent tissues and to distant organs. Much of this phenotypic progression of increased invasiveness depends largely on the activation of EMT. Activation of the epithelial–mesenchymal transition increases the abilities of colorectal cancer cells to migrate, invade and extravasate.

We then determined the expression of EMT markers vimentin, N‐cadherin, and E‐cadherin to confirm whether BMAL1 affected cell motility via the EMT pathway. We observed increases in vimentin and N‐cadherin expression in RKO/BMAL1 and SW480/BMAL1 cells, whereas E‐cadherin level was remarkably decreased (Figure 3E,F). E‐cadherin act as the “caretaker” of the epithelial phenotype and its loss is considered a critical feature of the EMT experience. Additionally, the nuclear accumulation of β‐catenin is an important hallmark of the EMT at the invasive of CRC. Our study indicated that β‐catenin was activated in BMAL1‐positive CRC cell lines. In contrast, BMAL1 silencing reduced the expression of the vimentin, β‐catenin, and N‐cadherin proteins (Figure 3G,H). Taken together, BMAL1 regulated the activation of EMT process and the migration and invasion behavious of colorectal cancer.

### 3.4 BMAL1 upregulates c‐Myc expression

**FIGURE 4:**
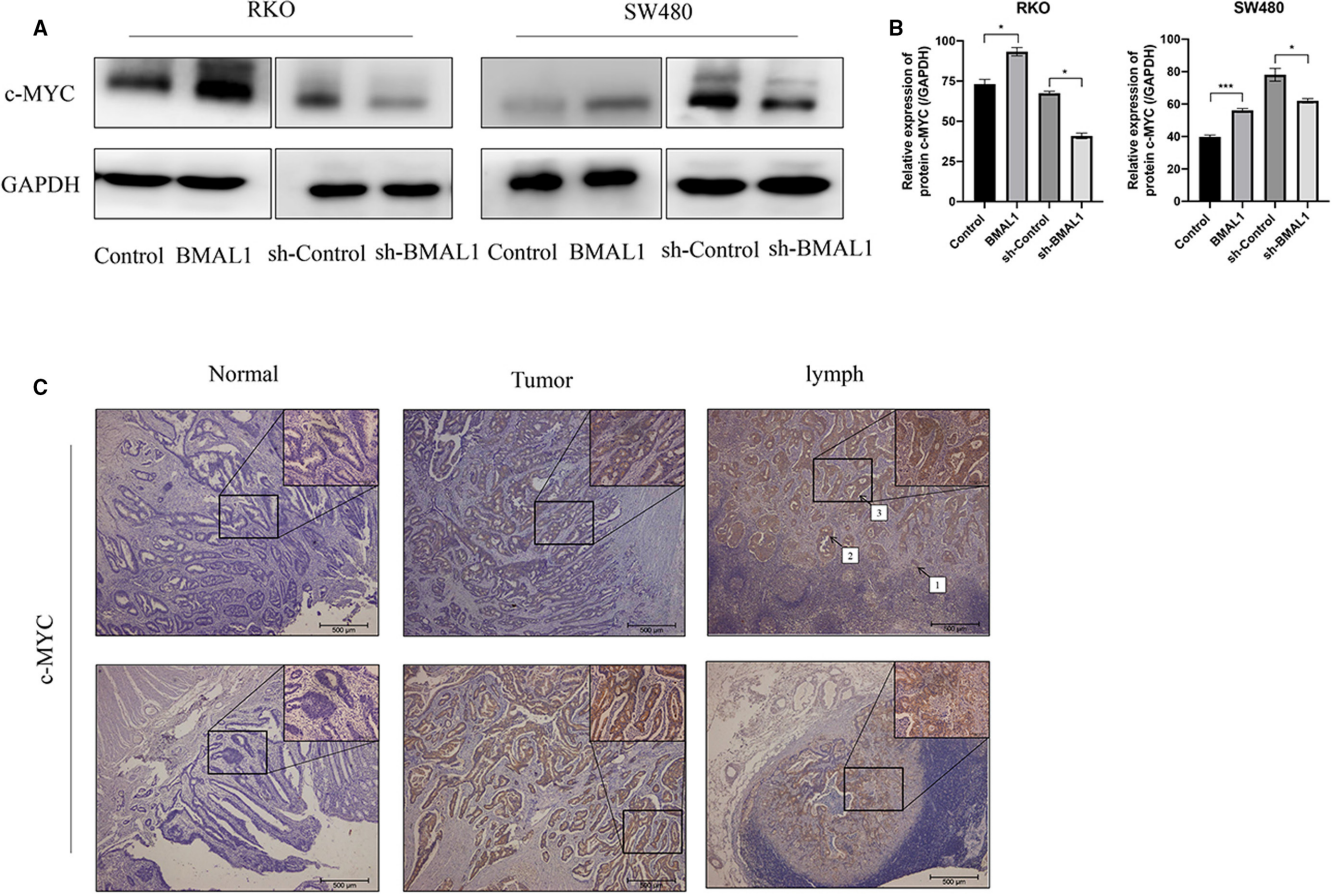
BMAL1 regulates c‐Myc expression. (A) BMAL1 activated c‐Myc protein expression in RKO/BMAL1 and SW480/BMAL1 cells, which detected by western blot. (B) Western blots were quantified by ImageJ densitometric analysis and normalized to controls. Data are expressed as mean ± SD (*n* = 3). (C) Immunohistochemical detection of c‐Myc in representative tumor samples, the adjacent normal tissue and lymph node from CRC of the indicated genotypes. 40× & 200× magnification. 1 = mild, 2 = moderate, 3 = strong. *p < 0.05, **p < 0.01 and ***p < 0.001.

We speculated that BMAL1 induces cell migration in vitro by moderating the expression of target genes. First, distant metastasis of CRC is driven by the EMT of cancer cells. c‐Myc is one of the most important regulators of the EMT that contributes to metastatic processes in CRC and other carcinomas. Additionally, β‐catenin was recently considered an important transcriptional activator of c‐Myc.^17^, ^18^, ^19^, ^20^ Upregulation of c‐Myc is a hallmark of colorectal cancer.^21^, ^22^, ^23^ More importantly, previous studies have implied that c‐Myc is also a target of aberrant clock gene expression.^24^ We hypothesized that c‐Myc may be essential for BMAL1 to promote cell metastasis and activate the EMT pathway. Therefore, c‐Myc expression was investigated in RKO/BMAL1 and SW480/BMAL1 cell lines using Western blotting. c‐Myc expression was significantly upregulated in BMAL1‐positive cell lines. In contrast, c‐Myc expression was remarkably decreased in BMAL1‐silenced cell lines (Figure 4A,B).

For further confirmation, we next explored whether the same results would be obtained in the clinical samples. We investigated BMAL1 and c‐MYC protein levels via IHC in clinical samples. Among the specimens from patients with colorectal cancer, 72.1% had c‐Myc overexpression (Figure 4C). The prevalence of c‐Myc overexpression was significantly higher in the BMAL1‐positive group than in the BMAL1‐negative group (*p* < 0.05). Interestingly, all patients in the BMAL1 overexpression group were c‐Myc‐positive (Table 2). Based on these results, we conclude that a high level of BMAL1 was associated with c‐Myc overexpression. All of the aforementioned findings support the role of c‐Myc as a potential target of BMAL1 that contributes to cell invasion and metastasis.

**TABLE 2:** BMAL1 and c‐Myc status in primary tumors in 68 patients with CRC

	c‐Myc		Total	*p‐*value
	Negative	Positive		
BMAL1				
Negative	19	11	30	
Positive	0	38	38	<0.01
Total	19	49	68	

### 3.5 BMAL1 activates c‐Myc through the MAPK signaling pathway

**FIGURE 5:**
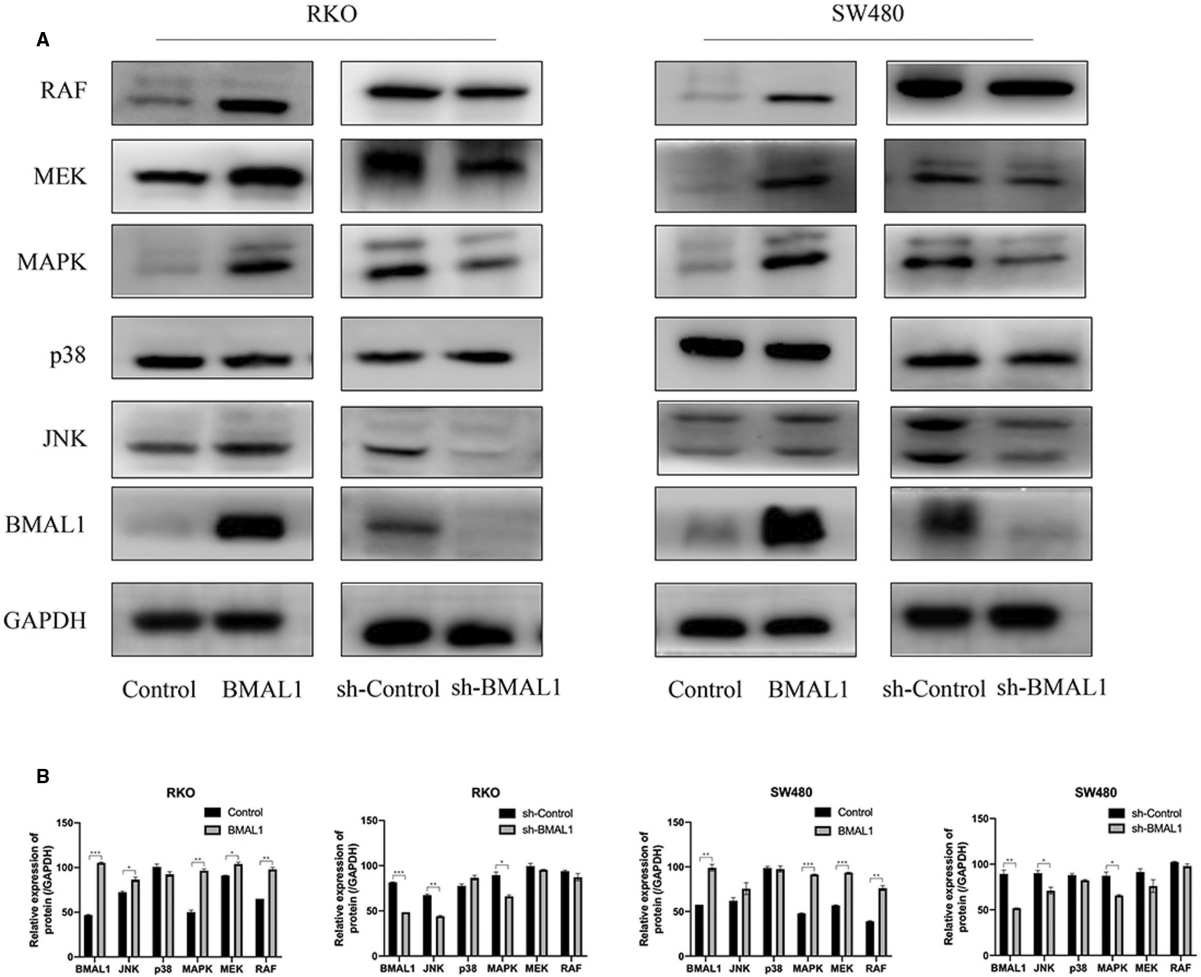
MAPK‐signaling pathway is involved in BMAL1‐induced migration. (A) BMAL1 activated MAPK‐signaling pathway in RKO/BMAL1 and SW480/BMAL1 cells. The related proteins in MAPK‐signaling pathway were detected by western blot. BMAL1 actived the RAF, P‐MEK, P‐ERK, JNK expression, while the P38 was not affected. (B) Data are expressed as mean ± SD (*n* = 3). *p < 0.05, **p < 0.01 and ***p < 0.001.

**FIGURE 6:**
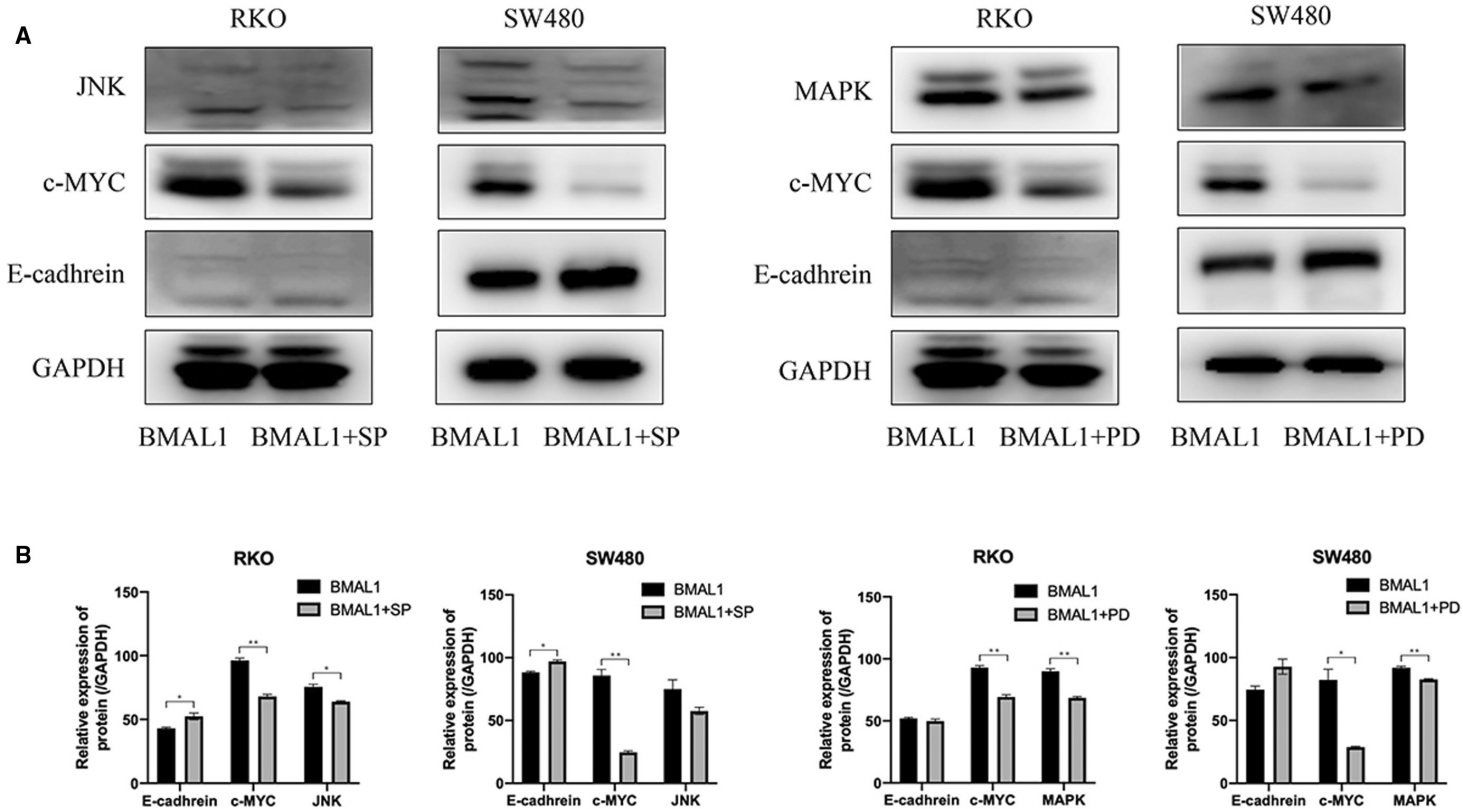
BMAL1 modulates the c‐Myc expression through ERK‐ and JNK‐dependent signaling pathway. (A) Inhibiting the MAPK pathway suppressed c‐Myc but increased E‐cadhrein expression in vitro using ERK1/2 (APExBIO PD98059, 10 μM) and JNK inhibitor (SP600125, 10 μM). (B) MAPK pathway inhibition markedly decreased the protein expression of c‐Myc. *p < 0.05, **p < 0.01 and ***p < 0.001.

From the above results, BMAL1 activates the target gene c‐Myc and the EMT pathway. Next, we would like to address how BMAL1 induces the EMT in CRC cells. To date, little is known about the effect of BMAL1 on intracellular signal transduction in CRC cells. As shown here, BMAL1 promoted the expression of c‐Myc. Therefore, we explored the potential relationship between these two genes and investigated several important signaling pathways. The MAPK pathway is not only a key regulator of the cell cycle distribution, survival, and drug sensitivity. It is also related to cell growth and migration. c‐Myc is one of the most important targets of the MAPK signaling pathway. The levels of MAPK pathway related molecules and their phosphorylated forms were detected to determine whether BMAL1 regulates the migration of CRC cells through this pathway. The results of Western blot analyses showed that the levels of RAF, p‐MEK, P‐ERK1/2, and P‐JNK (phosphorylated forms of MEK1/2, ERK1/2 and JNK) were significantly higher in RKO/BMAL1 and SW480/BMAL1 cells, but no changes were detected in P38 levels. In addition, significant changes in the levels of MAPK proteins were attenuated by BMAL1 silencing (Figure 5A,B). MAPK pathway antagonists were used to elucidate whether MAPKs, ERK, and JNK are linked to the BMAL1 stimulated c‐Myc expression. In the presence of ERK1/2 (APExBIO PD98059, 10 μM) and JNK inhibitors (SP600125, 10 μM), the overexpression of c‐Myc induced by BMAL1 was attenuated in RKO and SW480 cells, and cell migration was also dramatically decreased (Figure 6A,B), indicating that the ERK/JNK pathway mediated the upregulation of c‐Myc expression and increased cell migration by BMAL1. Hence, we speculated that BMAL1 promoted cell invasion via the ERK/JNK‐cMyc axis.

### 3.6 BMAL1 silencing inhibited the tumorigenicity of colorectal cancer cells in nude mice

**FIGURE 7:**
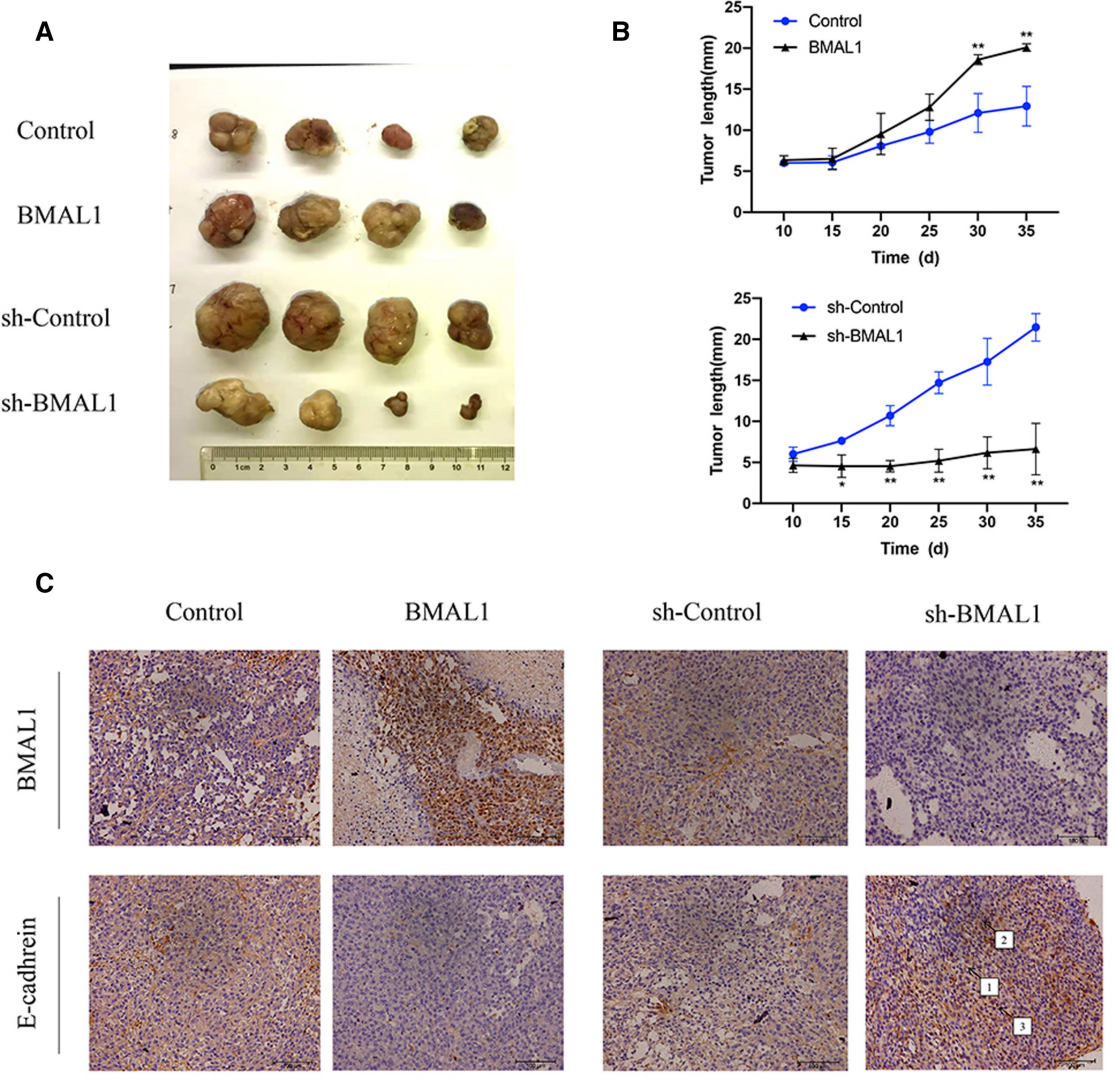
BMAL1 promotes tumorigenesis and tumor metastasis in mice. (A) BMAL1 knockdown significantly inhibited CRC proliferation in vivo. Cancer cells were injected into the nude mice. The tumors were resected from mice after 35 days. (B) At the indicated times, subcutaneous tumor size (mm) was measured with calipers (mean ± SD, *n* = 4). (C) Immunohistochemistry staining of BMAL1 and E‐cadhrein in xenograft tissues. 40× & 200× magnification. 1 = mild, 2 = moderate, 3 = strong. *p < 0.05, **p < 0.01 and ***p < 0.001.

Based on the results described above, silencing BMAL1 in colorectal cancer cell lines significantly inhibited cell proliferation and migration in vitro. A xenograft model was constructed to facilitate an investigation of these findings in vivo. RKO/Ctrl+ cells, RKO/BMAL1 cells, RKO/Ctrl‐ cells, and RKO/shBMAL1 cells were injected into the flanks of female nude mice. The tumors produced by the injection of RKO/BMAL1 cells were significantly larger and heavier than those in the control group after 5 weeks of management. RKO/BMAL1 cells exhibited markedly increased tumor growth in the subcutaneous xenograft model (*p* < 0.05, Figure. 7A,B).

Tissue sections (4‐μm‐thick slice) were cut from paraffin blocks generated after the tumors were measurable. In addition, the IHC staining and quantitative data also confirmed the results, and the percentage of the E‐cadherin staining was significantly increased in the RKO/shBMAL1 injection group (Figure 7C). Collectively, these results indicate that BMAL1 increased tumor cell growth and metastasis in nude mice.

## 4 DISCUSSION

CRC remains one of the most aggressive cancers due to its rapid recurrence and early metastasis. BMAL1 upregulation has been reported in various carcinomas, including colorectal cancer.^25^, ^26^, ^27^ Some studies reported that BMAL1 upregulation was associated with aggressive clinical phenotype and resistance to chemotherapy or targeted therapy.^28^ In contrast, others believed that BMAL1 was a protective factor for CRC,^29^ which might increase the sensitivity to oxaliplatin therapy.^30^ Our current study supports the role of BMAL1 in tumorgenicity and metastasis of CRC. To date, a few data on the mechanism of BMAL1 as a biomarker of CRC.

The EMT pathway is widely accepted to be a crucial biological process driving the invasion and metastasis of cancer cells. In this report, we provided evidence that increased BMAL1 expression in tumors promoted EMT‐like changes in colorectal cancer cell lines and lead to a dismal prognosis in patients with CRC. Previous studies have confirmed that circadian rhythm‐related genes have a significant influence on tumorigenesis, progression, and drug resistance. In our study, we measured BMAL1 expression levels in tissues of CRC patients. Clinical evidence suggests that BMAL1 overexpression is usually associated with poor outcome, and BMAL1 overexpression is more common in younger patients. Consistently, we investigated the levels of several typical EMT and proliferation markers, such as N‐cadherin, Ki67, β‐catenin, and vimentin. The expression of the proliferation‐related factor Ki67 significantly increased. Conversely, the levels of classical epithelial marker E‐cadherin remarkably decreased. Collectively, these evidences may prove that BMAL1 promotes the proliferate and migrate ability of colorectal cancer cell lines.

Our results reveal that BMAL1 activates the EMT pathway, which is the key and early stage in metastasis and invasion of CRC. As the ablation of E‐cadherin is a key feature of the EMT, cancer cells lose the connections between cells due to the decline of E‐cadherin. After EMT experience, cells gain greater motility to spread into surrounding or even distant tissues. Moreover, β‐catenin has been widely recognized as a major EMT marker that supported tumor attachment to the extracellular matrix.^31^, ^32^ EMT progress is not simply a binary process, but a highly complex program. Under normal conditions, β‐catenin is involved in the connecting between E‐cadherin and actin cytoskeleton. During EMT, β‐catenin separates from the E‐cadherin/β‐catenin complexes and accumulates in the cytoplasm. Specifically, disruption of the E‐cadherin/β‐catenin complex induces β‐catenin release and further avtivates Wnt/β‐catenin pathway. In addition to β‐catenin, c‐Myc is also the mediator of EMT and enhances the migration and invasion of CRC cells. Recent studies have shown that c‐Myc mutation or overproduction is related to circadian clock disorder.^33^, ^34^ The circadian clock gene Per2 has been suggested to lead to c‐Myc overexpression and an increased tumor incidence.^35^ Okazaki F showed that c‐Myc expression was controlled by the circadian clock in colon cancer cells.^36^ In this study, we studied the relationship between the BMAL1 and c‐Myc. We found that the level of c‐Myc changed with the expression of BMAL1. This association was confirmed in clinical patients treated at our hospital.

Additionally, the proto oncogene c‐Myc is one of the transcriptional targets of β‐catenin,which acts as a key downstream effector of WNT/β‐catenin signaling in several carcinogenesis processes.^37^ Our recent study evaluated the c‐Myc and β‐catenin expression in cancer cell lines, confirming the activation of β‐catenin/c‐Myc signaling in BMAL1‐positive colorectal cancer cells. In the BMAL1‐negative colorectal cancer cell lines, the expression of these two oncogenes was significantly decreased. Altogether, our data suggested that c‐Myc may be a target gene of BMAL1 in colon tumorigenesis. Next, we investigated the relationship between BMAL1 and c‐Myc in the CRC cells. c‐Myc was known as an important target gene of the MAPK pathway.^38^ MAPK signaling pathway consists of p38 MAPK, extracellular signal‐regulated kinase (ERK) and c‐Jun N‐terminal kinase (JNK). The relationship between MAPK pathway and cell proliferation and invasion in colorectal cancer has been fully studied.^39^ Therefore, we analyzed the expression of core proteins in these pathways to determine whether the function of BMAL1 in CRC depends on MAPK signaling pathways. The results showed that the levels of phosphorylated ERK1/2, JNK, and MEK1/2 in BMAL1 high expression group were significantly higher than BMAL1 silencing group. The invasiveness of the CRC cells was decreased after the administration of the corresponding inhibitors of MAPK signal pathway. Unexpectedly, p38 inhibition did not affect the BMALl‐induced migratory. Therefore, only the ERK/JNK MAPK pathway was activated by high BMAL1 expression in the present study. Next, we further examined the relationship between BMAL1‐induced MAPK pathway activation and c‐Myc expression. Stably transfected cells were treated with PD98059 or SP600125, then the MAPK and c‐Myc levels were analyzed by Western blotting. c‐Myc expression was significantly decreased. Moreover, the invasion activity was also suppressed. Therefore, the activation of ERK1/2 and JNK in MAPK pathway might play a critical role in BMAL1‐induced migration activation. c‐Myc is a key functional target gene modulated by BMAL1.

**FIGURE 8:**
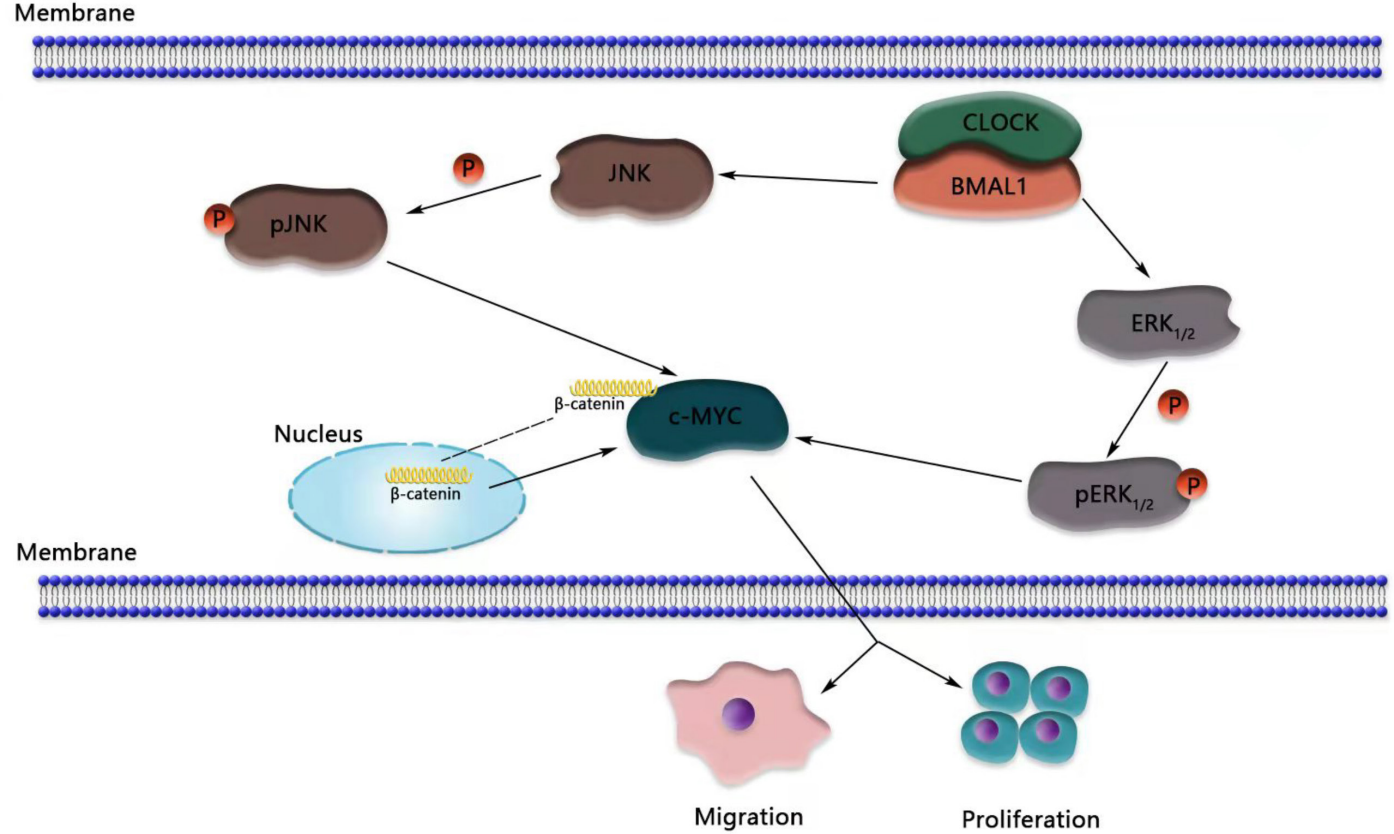
BMAL1 promotes colorectal cancer cells proliferation and migration via BMAL1/MAPK/c‐Myc pathway. This figure was created with BioRender.com.

This study documented the malignant biological functions of BMAL1, such as increasing colorectal cancer cell metastasis and proliferation. Meanwhile, we report for the first time that BMAL1 increased the expression of c‐Myc via the MAPK pathway in CRC. Inhibition of the ERK/JNK MAPK pathway suppressed the c‐Myc and EMT axes. We hypothesize that in BMAL1‐overexpressing cell lines, β‐catenin accumulates in the nucleus, and the MAPK pathway (ERK1/2 and JNK) is activated, resulting in increased c‐Myc expression and EMT pathway activation. Finally, decreased E‐cadherin expression leads to increased migration and ultimately promotes the metastasis of colorectal cancer (Figure 8).

In summary, our report specifically elucidated the interaction between BMAL1 and c‐Myc and illustrated that BMAL1 upregulates c‐Myc in CRC via activation of the MAPK‐signaling pathway.

## 5 CONCLUSION

Our report provides a new insight into the role of BMAL1 in colorectal cancer growth and metastasis. The BMAL1/MAPK/c‐Myc signaling loop in colorectal cancer may represent a novel mechanism to regulate colorectal cancer growth. Thus, targeting BMAL1 should be seen as a potential therapeutic opportunity for drug development to improve outcomes of patients with metastatic CRC.

## AUTHORS' CONTRIBUTIONS

LNS conceived and designed the study. LNS, WQZ, BJB and KKC performed the most part of experiments and wrote the manuscript. JHH and XWW provides help for the specific ideas of the article. The data were analyzed by YML. HBZ, XFH and SD reviewed the manuscript. All authors read and approved the manuscript.

## FUNDING INFORMATION

This work was supported by grants from the National Natural Science Foundation of China (grants no. 81703076 and 82072628) and the key research and development program of Zhejiang (grants no. 2022C03032).

## CONFLICT OF INTEREST

The authors declare that they have no competing interests.

## ETHICS APPROVAL AND CONSENT TO PARTICIPATE

This study was approved by the Ethics Committee of the Sir Run Run Shaw Hospital of Zhejiang University. Animal welfare and experimental procedures were carried out in accordance with the Guide for the Care and Use of Laboratory Animals and approved by the animal ethics committee of Zhejiang University (SRRSH201606035).

## CONSENT FOR PUBLICATION

All contributing authors agree to the publication of this article.

## Data Availability

The datasets generated and analyzed during the current study are available in the TCGA database (https://www.proteinatlas.org) (ENSG00000133794). All the data and material in this paper are available when requested.
